# TRPM7 Induces Mechanistic Target of Rap1b Through the Downregulation of miR-28-5p in Glioma Proliferation and Invasion

**DOI:** 10.3389/fonc.2019.01413

**Published:** 2019-12-17

**Authors:** Jingwei Wan, Alyssa Aihui Guo, Indrajit Chowdhury, Shanchun Guo, Jacqueline Hibbert, Guangdi Wang, Mingli Liu

**Affiliations:** ^1^Department of Microbiology, Biochemistry and Immunology, Morehouse School of Medicine, Atlanta, GA, United States; ^2^Department of Neurosurgery, The Second Xiangya Hospital, Central South University, Changsha, China; ^3^Department of Obstetrics and Gynecology, Morehouse School of Medicine, Atlanta, GA, United States; ^4^University of South Carolina SOM Greenville, Greenville, SC, United States; ^5^Department of Chemistry, Xavier University, New Orleans, LA, United States

**Keywords:** TRPM7, Rap1b, miR-28-5p, glioma, proliferation, invasion, prognosis

## Abstract

**Objectives:** Our previous findings demonstrate that channel-kinase transient receptor potential (TRP) ion channel subfamily M, member 7 (TRPM7) is critical in regulating human glioma cell migration and invasion. Since microRNAs (miRNAs) participate in complex regulatory networks that may affect almost every cellular and molecular process during glioma formation and progression, we explored the role of miRNAs in human glioma progression by comparing miRNA expression profiles due to differentially expressed TRPM7.

**Methods:** First, we performed miRNA microarray analysis to determine TRPM7's miRNA targets upon TRPM7 silencing in A172 cells and validated the miRNA microarray data using A172, U87MG, U373MG, and SNB19 cell lines by stem-loop RT-qPCRs. We next determined whether TRPM7 regulates glioma cell proliferation and migration/invasion through different functional domains by overexpressing wild-type human TRPM7 (wtTRPM7), two mutants with TRPM7's α-kinase domain deleted (Δkinase-DK), or a point mutation in the ATP binding site of the α-kinase domain (K1648R-KR). In addition, we determined the roles of miR-28-5p in glioma cell proliferation and invasion by overexpressing or under expressing miR-28-5p *in vitro*. Lastly, we determined whether a Ras-related small GTP-binding protein (Rap1b) is a target of miR-28-5p in glioma tumorigenesis.

**Results:** The miRNA microarray data revealed a list of 16 downregulated and 10 upregulated miRNAs whose transcripts are significantly changed by TRPM7 knock-down. Cell invasion was significantly reduced in two TRPM7 mutants with inactive kinase domain, Δkinase, and K1648R transfected glioma cells. miR-28-5p overexpression suppressed glioma cells' proliferation and invasion, and miR-28-5p under expression led to a significant increase in glioma cell proliferation and migration/invasion compared to that of the controls. miR-28-5p suppressed glioma cell proliferation and migration by targeting Rap1b. Co-transfection of siRap1b with miR28-5p inhibitor reduced the glioma cell proliferation and invasion, caused by the latter.

**Conclusions:** These results indicate that TRPM7's channel activity is required for glioma cell growth while the kinase domain is required for cell migration/invasion. TRPM7 regulates miR-28-5p expression, which suppresses cell proliferation and invasion in glioma cells by targeting Rap1b signaling.

## Introduction

High grade malignant gliomas, also called glioblastoma multiforme (GBM), the most common and aggressive primary brain tumor, are devastating, uniformly fatal tumors for which no effective therapies currently exist. We have reported that TRPM7 channels, a subfamily member of the transient receptor potential (TRP), regulate glioma stem cell (GSC) growth/proliferation through STAT3 and Notch signaling pathways (1). Because of traditional treatments' limited success in prolonging the overall survival of GBM patients, treatments that target genomic abnormalities, epigenetics, and epigenetic modulators have been attracting an increasing amount of attention for their influence in many tumors including glioma. miRNAs, one of the epigenetic effectors, reversibly regulate transcription through binding to complementary sequences of mRNA and silencing its translation into proteins. miRNAs are small noncoding RNAs, generally 19–22 nucleotides in length, that modulate protein-coding genes by binding to the 3'-untraslated region (UTR) of the target mRNA, therefore disrupting transcription (2–4). Many studies have shown that miRNA are expressed in a variety of human tumors and exert dramatic functions in human tumorigenesis and metastasis (5). miRNAs can function as either tumor suppressor by inhibiting the expression of oncogene or tumor promoter by reducing the expression of tumor suppressor gene (6, 7). While a specific miRNA may simultaneously regulate different targets, namely a given miRNA's upstream regulation can involve different regulators at different steps of mRNA biogenesis, a single protein target can be regulated by different miRNAs. Therefore, miRNAs participate in complex regulatory networks that affect almost every cellular and molecular processes in tumor initiation and progression (6). Since miRNAs have significant roles in human tumorigenesis and metastasis (5), identification of aberrantly expressed miRNAs is a crucial initial step in illustrating miRNA-mediated tumorigenic pathways.

Our in-depth data analysis from miRNA microarray data revealed a list of 16 downregulated and 10 upregulated miRNAs whose transcripts are statistically significant with fold changes >2 by TRPM7 knock-down in A172 glioma cells. Among these, the miRNA of hsa-miR-28-5p (miR-28-5p) has been shown to exert crucial influence on tumor growth and migration by modulating AKT (8), ERK (9), and IGF-1 (10) signaling pathways. Although oncogenic and invasive roles of TRPM7 (11, 12) and tumor-inhibitory role of miR-28-5p (13) have been reported in multiple studies, how TRPM7 regulates miRNA and downstream target genes are unclear. To seal the gap between TRPM7 and miRNA in the existing literature, we determined the changes in miRNA in response to reduced TRPM7 expression in glioma cells. The aim of the present study was to investigate the functional roles of miR-28-5p to elucidate the molecular mechanism of TRPM7's regulation of miR-28-5p in multiple glioma cell lines with different genomic mutational status. Our data revealed that miR-28-5p was inversely regulated by TRPM7 expression in glioma cells; overexpression of miR-28-5p significantly inhibited glioma cell growth, migration, and invasion; the gene encoding Ras-related protein Rap1b, positively correlated with TRPM7, is a promising candidate target gene of miR-28-5p and may serve as a predictive marker for the poor survival of glioma patients (14, 15). Our study elucidated the crucial roles of TRPM7/miR-28-5p/Rap1b axis in gliomagenesis.

## Materials and Methods

Antibody and reagents: Primary antibodies used included anti-TRPM7 (Abcam, Cat no. ab232455, Cambridge, MA) and anti-β-actin (Sigma-Aldrich, St. Louis, MO, Cat no. A3854). Rabbit polyclonal Rap1b (36E1, Cat no. 2326) and mouse HA (Cat no. 2367) antibodies were purchased from Cell Signaling Technology (Danvers, MA). All secondary antibodies used for Western blot were purchased from Calbiochem (La Jolla, CA).

### Plasmid, siRNA, and miRNAs

The wild-type human TRPM7 (wtTRPM7) or constructs in which the α-kinase domain was deleted (Δkinase) or rendered inactive with a point mutation in the ATP binding site of the α-kinase domain (K1648R) were provided by Dr. Carsten Schmitz, University of Colorado, Denver, CO. All constructs (wtTRPM7, Δkinase, K1648R) were tagged with a hemagglutinin (HA) at the N-terminal. Control scrambled siRNA (On-TARGETplus Non-targeting siRNA, Cat no. D-001810-01-05) and ON-TARGETplus SMARTpool siRNA (Cat no. L-005393-000005) targeting TRPM7 were purchased from Dharmacon (Lafayette, CO). Control scramble siRNA (Cat no. sc-37007) and siRNA targeting Rap1b (Cat no. sc-41854) were purchased from Santa Cruz Biotechnology (Santa Cruz, CA). The scrambled siRNAs, with no homology to any known sequence were used as controls. miR-28-5p mimic (Cat no. 4464066), miR-28-5p inhibitor (Cat no. 4464084), and control were purchased from Life Technology (Carlsbad, CA).

### Cell Culture

Human glioblastoma cell line, A172, was obtained from ATCC (Manassas, VA, USA). Other glioma cell lines, U87MG, U373MG, and SNB19, were kindly provided by Dr. Yancey G. Gillespie at the University of Alabama at Birmingham (UAB), Birmingham, AL. Human glioblastoma cell line SF767 was kindly provided by Dr. Hui-Kuo Shu at Emory University, Atlanta, Georgia. All cells were cultured in Dulbecco's Modified Eagle's Medium (DMEM, Sigma-Aldrich, St. Louis, MO) plus 10% fetal bovine serum (FBS), 50 units/ml penicillin, and 50 μg/ml streptomycin at 37°C.

### Transfection of siRNA and Expression of Wild-Type Human TRPM7 and Mutant Constructs in Glioma Cells

When the glioblastoma cells reached about 50–75% confluency, the appropriate amount of siRNAs specific to TRPM7, Rap1b, and control scramble siRNA with the final concentration of 100 nM, and miR-28-5p mimics, inhibitor, and controls with final concentration of 30 nM were transfected using Lipofectamine RNAiMAX reagent in serum free OptiMEM-1 medium (Invitrogen, Carlsbad, CA) according to the manufacture's instruction. After six hours of transfection, cells were grown in Dulbecco's Modified Eagle Medium (DMEM) containing 10% fetal bovine serum (FBS) (16, 17) further for 24–72 h as indicated in each experiment. 48 or 72 h post transfection, targets knockdown were assessed by RT-PCR or Western blot, respectively. Various glioma cells at 50–75% confluency were transfected with a pcDNA4/TO plasmid that allowed protein expression of wt hTRPM7 and hTRPM7 mutants for the α-kinase deletion or lacking of phosphotransferase activity by lipofectamine 3000 transfection reagent (Invitrogen, Carlsbad, CA) according to the manufacture's instruction. The transiently transfected glioma cells expressing wt hTRPM7, Δkinase, and K1648R hTRPM7 constructs were maintained in DMEM containing 10% FBS (16, 17) for further growth for 24–72 h. The overexpression of TRPM7 and its mutants were assessed by HA expression. All studies were done in triplicates.

### MTT Assay

All glioma cells were seeded at 1 × 10^4^ cells in 100 μl of medium per well into 96-well plates and were transfected with 100 nM specific siRNA or control using Lipofectamine reagent for indicated times. Ten micorliter of 3-(4,5-dimethylthiazol-2-yl)-2,5-diphenyltetrazolium bromide (MTT) reagent (Sigma-Aldrich, St. Louis, MO, the ratio of MTT reagent to medium is 1:10) was added into each well and incubated in the dark at 37°C for 2–4 h. Absorbance at 570 nm was measured using 690 nm as the reference using a CytoFluorTM 2300 plate reader.

### Cell Migration and Invasion Assay

The migration and invasion potential were assessed as previously described (1, 18). Briefly, cell culture chambers with 8 μm pore size of polycarbonate membrane filters (Corning, USA) were used for cell invasion assays with the filters pre-coated with matrigel (50 μl, 1.25 mg/ml). Each of the glioma cell lines were transfected with or without 100 nM siRNA for 48 h, harvested and seeded with 1% FBS medium in the upper chambers that were soaked in bottom chambers filled with 500 μl whole medium (DMEM and 10% FBS). After another 24 h of incubation at 37°C, matrigel and cells on the upper surface of the filter were wiped off thoroughly with a Q-tip. Cells attached on the lower surface of the membrane filters were fixed with 4% paraformaldehyde/PBS for 10 min and stained with 0.5% crystal violet/methanol for 10 min. The cells were then counted under light microscopy with 10x magnification in 3–4 random fields. Cell numbers under different treatments were normalized to the appropriate controls. Assays were done in triplicate of samples and were performed in two independent experiments.

### miRNA Microarray

TRPM7-siRNA was employed to selectively suppress the expression of TRPM7 channels in A172 cells. Then, total RNA was extracted using the miRNeasy Mini Kit (Qiagen, Valencia, CA) and was subjected to the human miRNA array assay by LC Sciences (Houston, TX, www.LCsciences.com) using MRA-1001B2 version miRHuman 22, containing 2,632 standard mature miRNA unique probes with 3 repeats, 50 controls (8–32 repeats) based on Sanger miRBase Release 22 (http://www.mirbase.org/). PUC2PM-20B and PUC2MM-20B are the control probes for quality controls of chip production, sample labeling, and assay conditions. Fold changes and *P*-values were calculated using Student's *t*-test. A *P* < 0.05 with a fold change >2.0 was considered to be a significant dysregulation. In-depth data analysis from miRNA microarray data showed a list of 16 downregulated and 10 upregulated miRNAs whose transcripts are statistically significant with fold changes >2 by TRPM7knock-down.

### Real-Time RT-PCR Analysis

Total RNA isolation, cDNA synthesis, and PCR amplification were performed as previously described (19). Cell pellets were stored in Trizol reagent and homogenized in fresh Trizol. Total RNA was isolated from cells using a miRNeasy Kit (Qiagen, Valencia, CA) and quantified using the Nanodrop N-1000 by Agilent Biosystems (Santa Clara, CA). Purified total RNA (0.75 μg) was reverse transcribed using iScript cDNA Synthesis Kit according to the manufacture's protocol (Bio-Rad Laboratories, Inc., Hercules, CA). Reverse transcription was performed by using random hexamers at 25°C for 5 min, 42°C for 30 min, and 85°C for 5 min. After diluting 10 times, the cDNA was then amplified using iQ SYBR Green Supermix (Bio-Rad Laboratories, Inc.) according to the manufacture's protocol under the following conditions: activation of the Taq DNA polymerase at 95°C for 3 min, 40 cycles at 95°C for 10 s (denaturation), and 61°C for 45 s (combined annealing and extension). The quantitative gene analysis utilized the CFX Connect Real Time PCR Detection System. Each condition was conducted in biological triplicates, and each individual biological replicate was amplified in technical triplicates. Relative expression for each gene was evaluated using the 2^−ΔΔ*Ct*^ Livak method, and GAPDH was used as the reference gene (20). We used the melting curve analysis to assess whether or not the intercalating dye qPCR assays have produced single, specific product. The single peak was observed for each specific gene, which represented as a pure single amplicon, indicating the specificity of each primer for each specific gene.

### Stem-Loop Pulsed Reverse Transcription: A Highly Sensitive RT-PCR Method for the Detection and Quantification of miRNAs

The miRNA validation was performed using stem-loop pulsed RT-PCR with some modifications as described before (21). The RT primer for miR-28-5p reverse transcription, forward and reverse primers for RT product amplification were designed based on miR-28-5p's sequence: AAGGAGCUCACAGUCUAUUGAG (http://www.mirbase.org/). For each reaction, “no RNA” master mix comprised of 10 mM dNTP, 5 μM RT primer (see Table 1), and appropriate water, was heated at 65°C for 5 min and incubated on ice for 2 min. Then, the “no RNA” master mix was combined with RT master mix containing first-strand buffer, 0.1M DTT, 4 units RNaseOUT, and 50 units of SuperScript III reverse transcriptase. Then the pulsed RT was performed under the following conditions: load thermal cycler and incubate for 30 min at 16°C, pulsed RT of 60 cycles at 30°C for 30 s, 42°C for 30 s and 50°C for 1 s, and incubate at 85°C for 5 min to inactivate the reverse transcriptase. Finally, the RT product was amplified using iQ SYBR Green Supermix (Bio-Rad) as described above.

**Table 1:** List of primers used in the study.

**Primer set**	**Forward 5^**′**^-3^**′**^**	**Reverse 5^**′**^-3^**′**^**
TRPM7	CTTTGACCAAGAGGGAATGTG	GACCAAGCGACCACAAAAAC
Rap1b	TGCTTGAAATCTTGGATACTGC	GTGGACTGTGCTGTGATGGA
GAPDH	GAAGGTGAAGGTCGGAGTC	GAAGATGGTGATGGGATTTC
SNOD48	AGTGATGATGACCCCAGGTAA	AGAGCGCTGCGGTGAT
SNOD44	CCTGGATGATGATAAGCAAATG	TTAGAGCTAATTAAGACCTTCATGTTC
SNOD47	ATGATGTAATGATTCTGCCAAA	CCTCAGAATCAAAATGGAACG
U6	CAAGGATGACACGCAAATTC	AAAAATGGAACGCTTCACGA
miR28-5p RT primer	GTCGTATCCAGTGCAGGGTCCGAGGTATTCGCACTGGATACGACCTCAAT	
miR28-5p	GTATACAAGGAGCTCACAGTC	GTGCAGGGTCCGAGGT

### Western Blotting

Cells were lysed with lysis buffer (50 mM HEPES, 150 mM NaCl, 1.5 mM MgCl2, 1 mM EGTA, 10% glycerol, 1% Nonidet P-40, 100 mM NaF, 10 mM sodium pyrophosphate, 0.2 mM sodium orthovanadate, 1 mM phenylmethylsulfonyl fluoride, 10 μg/ml aprotinin, and 10 μg/ml leupeptin). Samples were separated by SDS/PAGE, and separated proteins were transferred to nitrocellulose membranes and identified by immunoblotting. Primary antibodies were obtained from commercial sources and were diluted at the ratio of 1:1,000 according to manufacturer's instruction. Blots were developed with Supersignal Pico or Femto substrate (Pierce). A densitometric analysis of the bands was performed with the ImageQuant program (Bio-Rad).

### Bioinformatics Analysis

Kaplan-Meier analysis of overall survival according to the TRPM7, Rap1b mRNA expression and Pearson correlation between TRPM7 and Rap1b were obtained from microarray analysis on 454 glioma patients in the TCGA data set (http://www.betastasis.com/glioma/tcga_gbm/). *P*-value is based on log rank test.

### Statistical Analysis

The results obtained in this work were expressed as mean ± SD of at least 2 independent experiments done in triplicate. Paired Student *t*-test or one-way ANOVA tests were performed for data analysis, and significant difference was defined as *P* < 0.05.

## Results

### TRPM7 Regulates Glioma Cell Proliferation and Migration/Invasion Through Different Functional Domains

**Figure 1:**
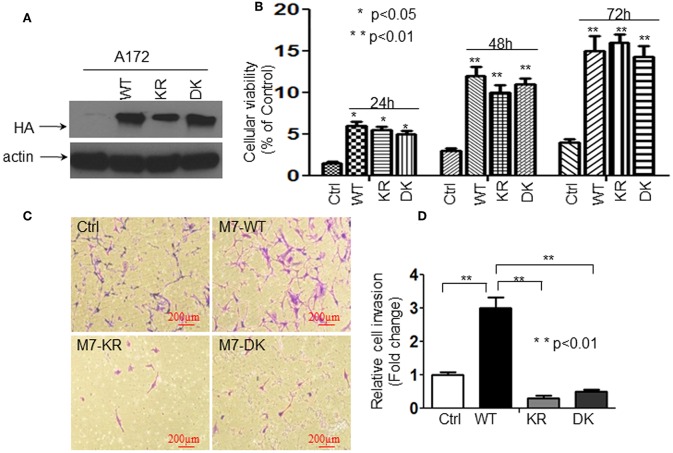
The channel activity of TRPM7 is required for cell growth while the kinase domain is required for cell migration/invasion in A172 cells. A172 cells were transfected with (a) 5 μg of wild-type human TRPM7 (wtTRPM7 or M7-WT or WT); (b) constructs in which the α-kinase domain was deleted (Δkinase or M7-DK or DK); (c) rendered inactive with a point mutation in the ATP binding site of the α-kinase domain (K1648R, or M7-KR or KR) along with control vector (Ctrl); all the cells were allowed to grow from 24 to 72 h as indicated. **(A)** The transfection efficiency was assessed by detecting HA-TRPM7 with an anti-HA antibody by Western blot. A172 cells with Δkinase (M7-DK) and K1648R (M7-KR) mutants expressed HA-TRPM7 protein, the mutants expressed the equivalent levels of HA-TRPM7 expression as that of wt TRPM7 expression; while there is undetectable HA-TRPM7 in control. **(B)** The effects of TRPM7 on glioma cell proliferation were determined using MTT assays. Cell numbers were counted every 24 h for 3 days post-transfection with wtTRPM7, M7-DK, or M7-KR as well as the control. The values represent a fold change relative to controls and are mean ± SD of triplicated samples performed in three independent experiments. **(C)** For transwell invasion assays, each transfected A172 cells were grown for 48 h, then cells were harvested and seeded with 1% FBS medium in the upper chambers which were soaked in bottom chambers filled with 500 μl whole medium containing 10% FBS. The cells were then subjected to grow for 24 h during the invasion assay. A representative experiment of microscopy images (x10) shown the invasive cells on Transwell assay. Photomicrographs were taken at the magnification of 10x. **(D)** The cell numbers were counted on the bottom of Matrigel-coated transwell chamber at 72 h post-transfection. The data were represented as fold changes relative to controls and are the mean ± SD of triplicate samples performed in two independent samples (**P < 0.01, Student *t*-test).

We have reported that the activation of TRPM7 channels plays an important role in the growth and proliferation of human glioma cells (1). In the current study, we further investigated whether or not changes in glioma cell proliferation and migration might be caused by channel domain-mediated and/or kinase domain-mediated TRPM7 activation. To this end, A172 cells were transfected with (a) 5 μg of wild-type human TRPM7 (wtTRPM7 or M7-WT); (b) constructs in which the α-kinase domain was deleted (Δkinase or M7-DK) or rendered inactive with a point mutation in the ATP binding site of the α-kinase domain (K1648R, or M7-KR); all the cells were allowed to grow from 24 to 72 h as indicated. Note that *in vitro* autophosphorylation assays have been done to confirm that the phosphotransferase activity of the M7-WT channels are easily detectable and much reduced in the absence of mutant channel activities under standard conditions by Dr. Carsten Schmitz's group who provided us the constructs (17). The effects of TRPM7 on glioma cell proliferation and invasion were determined using MTT assays and transwell invasion assays, respectively. As shown in Figure 1A, A172 cells with Δkinase (M7-DK) and K1648R (M7-KR) mutants expressed the TRPM7 protein, as demonstrated by Western blot, in which TRPM7 expression at the lysate level was similar to that of wtTRPM7 expression (Figure 1A). The cells overexpressing wt TRPM7 (M7-WT), M7-KR, or M7-DK grew significantly more (*P* < 0.05) than the cells transfected with control (Figure 1B) in a time-dependent manner from 24 to 72 h. Cell proliferation was not significantly changed when cells were transfected with Δkinase or K1648R compared to those transfected with wt TRPM7, which indicates that TRPM7 channel, rather than kinase activity, is required for A172 cell growth (Figure 1B). Cell invasion was increased up to 3 times in A172 cells transfected with wt TRPM7 compared to that of the control; by contrast, the cell numbers that penetrated the Matrigel-coated transwell were significantly reduced in cells transfected with Δkinase (M7-DK) to 30% or with K1648R (M7-KR) to 50% at 72 h compared to control, indicating that TRPM7 kinase activity is required for changes in cell migration and invasion (Figures 1C,D). These results suggest that the channel activity of TRPM7 is required for cell growth while the kinase domain is required for cell migration/invasion.

### TRPM7 Negatively Regulates miR-28-5p in Glioma Cells

#### The Expression Levels of hsa-miR-26b-5p, hsa-miR4530, and hsa-miR-28-5p Were Significantly Changed by TRPM7 Knock-Down

**Figure 2:**
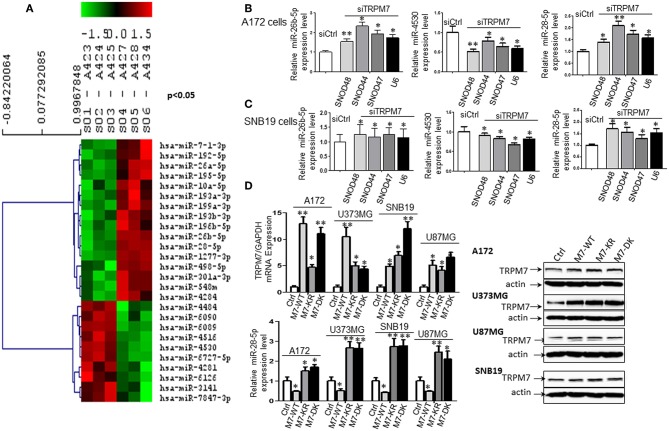
TRPM7 negatively regulates miR-28-5p in glioma cells. **(A)** Total RNA from biological triplicate samples, either with scramble control (siCtrl, A423, A424, and A425) or siTRPM7 (A427, A428, and A434), were extracted and then parallelly subjected to human miRNA array assay. In-depth data analysis from miRNA microarray data resulted in a list of 16 downregulated and 10 upregulated miRNAs whose transcripts are statistically significant with fold changes greater than 2 by TRPM7 knock-down. **(B,C)** Validation of miRNA array by siTRPM7 by qPCR. miR-26b-5p, miR-4530, and miR-28-5p were validated by real-time miRNA quantification using stem-loop RT-PCR in glioma cell lines A172 **(B)** and SNB19 **(C)**. A set of endogenous RNAs SNOD48, SNOD44, SNOD47, and U6 served as endogenous controls in real-time miRNA quantification to normalize miRNA gene expression. **(D)** Validation of miRNA array by overexpressing TRPM7. The constructs of wt hTRPM7 (M7-WT), Δ kinase (M7-DK), K1648R (MT-KR), and control (Ctrl) were introduced into and expressed in glioma cell lines A172, U373MG, SNB19, and U87MG for 48 h. Transfection efficiency was determined by TRPM7 mRNA expression detected by qRT-PCR. GAPDH was used as an internal control to monitor the presence of amplified cDNA in samples (upper left). Western blots by overexpression of individual TRPM7 constructs confirmed the data from qRT-PCR, which showed significantly increased TRPM7 in M7-WT, M7-KR, and M7-DK compared to the control (**D**, right). The levels of miR-28-5p were determined by stem-loop RT-PCR (lower left). All the data were represented as fold changes relative to the appropriate controls and are the mean ± SD of triplicated samples performed in three independent experiments. (*P < 0.05, **P < 0.01).

To test whether or not miRNAs are regulated by TRPM7 expression, 100 nM of siRNAs specific to TRPM7 (siTRPM7) or control scramble siRNA (siCtrl) were transfected into A172 cells for 72 h for specifically selective suppression of TRPM7 channel expression. Total RNA from triplicate biological samples, either with scramble control (siCtrl, A423, A424, and A425) or siTRPM7 (A427, A428, and A434) (Figure 2A), were extracted and parallelly subjected to human miRNA array assay by LC Sciences (Houston, TX, www.lcsciences.com). In-depth data analysis from miRNA microarray data revealed 16 downregulated and 10 upregulated miRNAs whose transcripts are statistically significant with fold changes >2 by TRPM7 knock-down (Figure 2A). Among them, microRNAs hsa-miR-26b-5p (miR-26b-5p), hsa-miR-4530 (miR-4530), and hsa-miR-28-5p (miR-28-5p) have been shown to exert crucial influence on tumor growth and migration by modulating AKT (8), ERK (9), and IGF-1 (10) signaling pathways. In our case, hsa-miR-26b-5p, hsa-miR-4530, and hsa-miR-28-5p were significantly changed by TRPM7 knock-down.

#### Validation of miRNA Array Results by siTRPM7

Next, miR-26b-5p, miR-4530, and miR-28-5p were further validated by real-time miRNA quantification using stem-loop RT-PCR (21–27) in glioma cell lines A172 and SNB19 (Figures 2B,C). A set of endogenous RNAs SNOD48, SNOD44, SNOD47, and U6 served as endogenous controls in real-time miRNA quantification to normalize miRNA gene expression. miR-26b-5p and miR-28-5p were significantly upregulated while miR-4530 was significantly downregulated upon TRPM7 silencing by siTRPM7 in both A172 and SNB19 cells as compared to each of their miRNA endogenous controls (^*^P < 0.05, ^**^P < 0.01, Student *t*-test). The data indicated that the expressions of hsa-miR-26b-5p, hsa-miR-4530, and hsa-miR-28-5p in the two cell lines are consistent with those of miRNA microarray data. Since miRNA's discovery decades ago, miR-26-5p, together with miR-21-5p and miR-30-5p was only detected in breast cancer and may not have significant roles in glioma; while miR-28-5p was found to be important in multiple malignancies including glioma. Therefore, we chose miR-28-5p to further elucidate the TRPM7-mediated pathways in glioma.

#### Validation of miRNA Array Results by Overexpressing TRPM7

The constructs of wt hTRPM7, Δ kinase, and K1648R were introduced into and expressed in glioma cell lines A172, U373MG, SNB19, and U87MG for 48 h. Total RNA and protein lysate were isolated, and TRPM7 levels were detected by qRT-PCR. GAPDH was used as an internal control to monitor the presence of amplified cDNA in samples. Optimum mRNA expression of wt hTRPM7 and the mutants, Δkinase and K1648R, was observed after 48 h. Overexpression of TRPM7 by M7-WT, M7-KR, and M7-DK dramatically increased TRPM7 mRNA as compared to that of the pcDNA4.2 control (Figure 2D, upper left, *P < 0.05, **P < 0.01, Student *t*-test) in each of four cell lines, indicating the system's high transfection efficiency. Western blots by overexpression of individual TRPM7 constructs for 72 h confirmed the data from qRT-PCR, which showed significantly increased TRPM7 in M7-WT, M7-KR, and M7-DK compared to the control (Figure 2D, right). Unlike proteins that showed consistent expression of M7-KR and M7-DK as M7-WT across the cell lines, there are varying expression at mRNA levels of M7-WT and M7-KR and M7-DK. This indicates that transcription levels are frequently not reflected at the protein level due to protein instability and lower rate of mRNA transcription compared to protein translation in mammalian cells (28). The levels of miR-28-5p were determined by stem-loop RT-PCR. The results showed that the overexpression of wt hTRPM7 decreased miR-28-5p expression by 53, 50, 57, and 51% in A172, U373MG, SNB19, and U87MG, respectively; on the other hand, K1648R mutants increased miR-28-5p expression by 1.5, 2.67, 2.74, and 2.45 times. Δ kinase mutants exhibited similarly increased miR-28-5p expression pattern as K1648R mutants did by 1.7, 2.64, 2.78 and 2.1 times, respectively (Figure 2D, lower left). These results suggest that wild type TRPM7 may have different functions from its mutants.

### miR-28-5p Inhibits Glioma Cell Proliferation and Invasion

**Figure 3:**
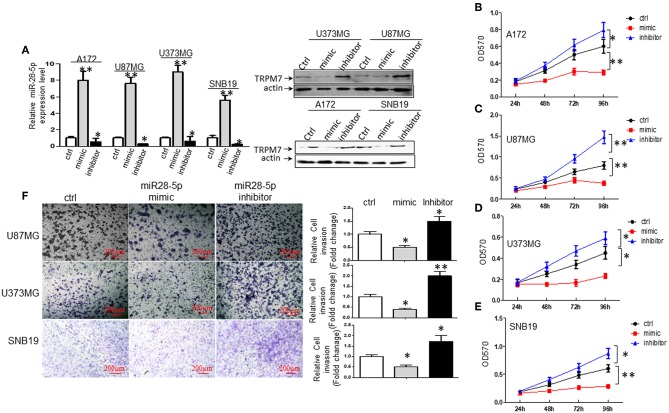
miR-28-5p inhibits glioma cell line proliferation and invasion. **(A)** miR-28-5p mimics, miR-28-5p inhibitors and miR-NC (or ctrl) were transfected into A172, U87MG, U373MG, and SNB19 glioma cells, and stem-loop RT-PCR was conducted to examine the levels of miR-28-5p expression 72 h post-transfection with miR-28-5p mimics, miR-28-5p inhibitors and miR-NC. Left: miR-28-5p expression was significantly increased in A172, U87MG, U373MG, and SNB19 transfected with miR-28-5p mimics compared to the expression levels in cells transfected with miR-NC, miR-28-5p was significantly decreased in the above four glioma cell lines transfected with miR-28-5p inhibitors compared to the expression levels in cells transfected with miR-NC (*P < 0.05, **P < 0.01, Student *t*-test). The data were presented as the fold changes relative to the corresponding controls and are mean ± SD of triplicate samples performed in three independent experiments. Right: TRPM7 protein expression levels were checked simultaneously when overexpressing miR-28-5p mimics and inhibitors, and the negative correlation between TRPM7 and miR-28-5p was confirmed. **(B–E)** Representative experiment of the proliferation effect of miR-28-5p in A172, U87MG, U373MG, and SNB19 glioma cell lines assayed by MTT. Absorbance was measured every 24 h for 4 days post-transfection with miR-NC, miR-28-5p mimics and miR-28-5p inhibitors. miR28-5p mimics significantly decreased cell proliferation in glioma cell lines; while miR-28-5p inhibitors significantly increased cell proliferation in glioma cell lines (*P < 0.05, **P < 0.01, Student *t*-test). The data were presented as the fold changes relative to the corresponding controls and are mean ± SD of triplicate samples performed in three independent experiments. **(F)** A Matrigel transwell invasion assay, using U373MG, SNB19, and U87MG cell lines that were transiently transfected with miR-NC, miR-28-5p mimics, and miR-28-5p inhibitors were conducted exactly same as described in Figures 1C,D. The cell numbers were counted on the bottom of Matrigel-coated transwell chamber at 72 h post-transfection. A representative experiment was shown. The ectopic expression of miR-28-5p inhibited the number of invaded cells while inhibition of miR-28-5p expression increased the invasion compared to the controls. The data were represented as fold changes relative to controls are the mean ± SD of triplicate samples performed in two independent samples (*P < 0.05, **P < 0.01, Student *t*-test). Photomicrographs were taken at 10x magnification.

To understand the biological effect of miR-28-5p on the proliferation of glioma cells, miR-28-5p expression was manipulated by transfecting A172, U87MG, U373MG, and SNB19 cells with either miR-28-5p mimics, inhibitors, or controls (ctrl or miR-NC) for 72 h to increase or decrease miR-28-5p expression levels. The stem-loop RT-PCR assay confirmed that miR-28-5p was significantly increased in A172, U87MG, U373MG, and SNB19 cells that were transfected with miR-28-5p mimics as compared to the expression levels in cells transfected with miR-NC (Figure 3A, left). Similarly, miR-28-5p was significantly decreased in the above four glioma cell lines that were transfected with miR-28-5p inhibitors as compared to the expression levels in cells transfected with miR-NC (Figure 3A, left). We assessed TRPM7 protein expression levels simultaneously when overexpressing miR-28-5p mimics and inhibitors by Western blot, and we confirmed the negative correlation between TRPM7 and miR-28-5p (Figure 3A, right).

We then examined the effects of miR-28-5p on glioma cell growth using MTT assay. As shown in Figures 3B–E, the overexpression of miR-28-5p by transient transfection of miR-28-5p mimics dramatically inhibited the growth rate of A172, U87MG, U373MG, and SNB19 glioma cell lines, where cell proliferation decreased in A172 (Figure 3B), U87MG (Figure 3C), U373MG (Figure 3D), and SNB19 (Figure 3E) by a maximum of 51.7, 52.5, 48.9, and 53.3% on day 4, respectively. In contrast, reduced miR-28-5p expression by miR-28-5p inhibitor augmented the growth of the four glioma cell lines above, where cell proliferation increased in A172 (Figure 3B), U87MG (Figure 3C), U373MG (Figure 3D), and SNB19 (Figure 3E) by a maximum of 32.0, 83.8, 31.1, and 45% on day 4, respectively. Next, we evaluated the possible roles of miR-28-5p in glioma cells invasion. Matrigel transwell invasion assays were performed on U87MG, U373MG, and SNB19 cell lines that were transiently transfected with miR-NC, miR-28-5mimics, and miR-28-5p inhibitors, and the results showed that the ectopic expression of miR-28-5p inhibited the number of invaded cells by 50, 60, and 40%, respectively as compared with that of the controls; on the other hand, miR-28-5p inhibitors enhanced the number of invaded cells by 50, 200, and 80%, respectively (Figure 3F).

### TRPM7 Positively Regulated Rap1b Expression in Glioma Cells

**Figure 4:**
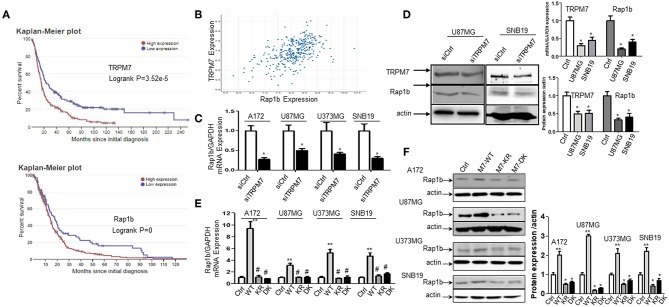
TRPM7 positively regulated Rap1b mRNA and protein expression in glioma cells. **(A)** Kaplan-Meier survival curve for the overall survival of glioma patients. The bioinformatics analysis from Rembrandt showed a high expression level of TRPM7 and Rap1b are unfavorable prognostic biomarkers of overall survival (*p* = 3.52e−5 for the former, *p* = 0 for the latter, logrank tests). The data in upper panel of **(A)** showed that glioma patients with high TRPM7 expression (*n* = 165) have short overall survival compared to those with low TRPM7 expression (*n* = 164). The glioma patients with high Rap1b (lower panel) predict the poor prognosis of glioma patients as well (**A**, lower panel). **(B)** By Pearson correlation analysis via TCGA (http://www.betastasis.com/glioma/tcga_gbm/), a clearly direct correlation was observed between TRPM7 and Rap1b expression levels in 356 glioma patients with both genes are detectable (*r* = 0.515). **(C–F)** To confirm this correlation, we transfected A172, U87MG, U373MG, and SNB19 cells either with synthesized specific small interfering RNAs (siTRPM7) targeting TRPM7 mRNA to decreasing TRPM7 expression or with TRPM7 expression vector along with its mutants Δ kinase and K1648R to increase the TRPM7 expression. The transfection efficiency of TRPM7 knock-down and overexpression were examined by qPCR [see Figure 2D and **(D)**, upper right] and Western blot (**D** left and lower right). The lower right panel of **(D)** is the densitometry analysis of the bands performed using the ImageQuant program. The results showed that the inhibition of TRPM7 expression by siTRPM7 markedly reduced Rap1b expression at both mRNA in all four cell lines (**C**, *P < 0.05, Student *t*-test) and protein levels in U87MG and SNB19 cells **(D)**. While overexpression of TRPM7 dramatically enhanced the expression of Rap1b at both mRNA (**E**, **P < 0.05 compared to control-Ctrl, ^#^*P* < 0.05 compared to wild type-WT, one-way ANOVA) and protein levels **(F)** in all four glioma cell lines. The right panel of **(F)** is the densitometry analysis of the bands performed using the ImageQuant program. Again, the mutants Δ kinase and K1648R exhibited a noticeable difference in Rap1b expression pattern at both mRNA and protein levels compared to that of wt TRPM7, which reflect differences in their functional roles. The data are expressed as the fold changes relative to the corresponding controls and are the mean ± SD of triplicated samples preformed in three independent experiments **(C–D)**. All the data represent one of the three independent experiments with similar results **(D–F)**.

The biological roles of miRNAs in human tumors depend on their specific targets; therefore, we searched the literature for potential targets of miR-28-5p. We found 52 publications that related miR-28-5p with human tumors, with most of them serving as a tumor suppressor. Several important tumor-related genes were defined as the direct target for miR-28-5p, such as AKT in gastric cancer (8), N4BP1 in ovarian cancer (29), IL-34 (30) and IGF-1 (10) in hepatocellular carcinoma, and Rap1b in renal cell carcinoma (31). Among these, Rap1b has drawn our attention for the following reasons. First, Rap1b has been found to play a role in the glioma cell proliferation and migration (15, 32). Second, the bioinformatics analysis from TCGA (http://www.betastasis.com/glioma/tcga_gbm/) indicated both TRPM7 (Figure 4A, upper panel) and Rap1b (Figure 4A, lower panel) predict glioma patients' poor prognosis. Third, TRPM7 and Rap1b mRNA expression levels are correlated by Pearson's correlation analysis (*r* = 0.515) (Figure 4B). To confirm this correlation, we transfected A172, U87MG, U373MG, and SNB19 cells either with synthesized specific small interfering RNAs (siTRPM7) targeting TRPM7 mRNA to decreasing TRPM7 expression or with TRPM7 expression vector along with its mutants K1648R and Δ kinase to increase the TRPM7 expression. The transfection efficiency of TRPM7 knock-down and overexpression were examined by qPCR (Figure 2D, upper left and Figure 4D, upper right) and Western blot (Figure 4D, left and lower right). The results indicated that the inhibition of TRPM7 expression by siTRPM7 markedly reduced Rap1b expression at both mRNA (Figures 4C,D, upper right) and protein levels (Figure 4D, left and lower right); while the overexpression of TRPM7 dramatically enhanced the expression of Rap1b at both mRNA (Figure 4E) and protein levels (Figure 4F) in all four glioma cell lines. Again, the mutants K1648R and Δ kinase exhibited a noticeable difference in Rap1b expression pattern at the protein levels compared to that of wt TRPM7, which reflect differences in their functional roles (Figure 4F).

### Rap1b Elevates Glioma Cell Proliferation and Invasion

**Figure 5:**
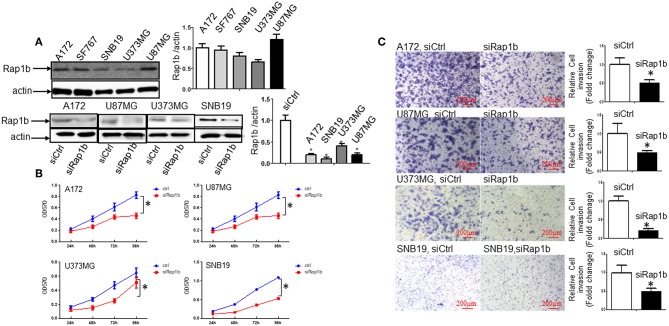
Rap1b elevates glioma cell proliferation and invasion. **(A)** All the glioma cell lines A172, SF767, SNB19, U373MG, and U87MG expressed the Rap1b protein (upper panel). We then transfected siRap1b along with the control into the in A172, U87MG, U373MG, and SNB19 cells, the efficiency of the transfection was detected by the expression of total Rap1b protein, as examined by Western blot. Rap1b siRNA markedly inhibited Rap1b expression (lower panel). The corresponding densitometry analysis for each Western blot performed using the ImageQuant program are on the right sides correspondingly. **(B,C)** Rap1b elevates glioma cell proliferation and invasion. MTT and cell invasion assay were conducted to examine the effects of Rap1b on the proliferation and invasion after transfecting A172, U87MG, U373MG, and SNB19 with synthesized specific small interfering RNAs (siRap1b) targeting Rap1b mRNA. The results showed that transfection with siRap1b resulted in a significant decrease in proliferation **(B)** and invasion (**C**, Photomicrographs were taken at 10x magnification) compared with the respective siRNA control groups in the four glioma cell lines. These results suggested that Rap1b increases glioma cell line proliferation and invasion, and Rap1b may perform tumor promoting roles in glioma growth and metastasis [*P < 0.05, one-way ANOVA for proliferation assay in **(B)**, Student *t*-test for invasion assay in **C**]. All data are from triplicate samples performed in two different independent experiments.

We first determined whether or not Rap1b protein, a Ras-related small GTP-binding protein that acts as GTPase in several signaling cascades, is expressed in glioma cells. As shown by Western blot, all of the glioma cell lines A172, SF767, SNB19, U373MG, and U87MG expressed the Rap1b protein (Figure 5A, upper panel). To confirm whether or not Rap1b functions as an oncogene in glioma, we assessed the effects of Rap1b on glioma cell proliferation and invasion *in vitro* using MTT assay or Matrigel transwell invasion assay after transfecting A172, U87MG, U373MG, and SNB19 with synthesized specific small interfering RNAs (siRap1b) targeting Rap1b mRNA. The efficiency of the transfection was detected by the expression of total Rap1b protein, as examined by Western blot. As shown in Figure 5A, Rap1b siRNA markedly exhibited the inhibition of Rap1b expression (Figure 5A, lower panel). Next, MTT and cell invasion assay were conducted to examine the effects of Rap1b downregulation on the proliferation and invasion in A172, U87MG, U373MG, and SNB19, respectively. The results showed that transfection with siRap1b resulted in a significant decrease in proliferation in an approximate time-dependent manner when compared with the respective siRNA control groups in the four glioma cell lines; the cell growth viability was reduced by a maximum of 44.4% at 96 h in A172 cells, 37.4% at 96 h in U87MG cells, 46.5% at 72 h in U373MG cells, and 54.3% at 72 h in SNB19 cells (Figure 5B). The number of invasive cells crossed the Matrigel transwell was dramatically reduced by 40, 50, 80, and 55% in A172, U87MG, U373MG, and SNB19 cells at 72 h post-transfection, respectively (Figure 5C). Taken together, these results suggest that Rap1b increases glioma cell line proliferation and invasion, and promotes glioma tumorigenesis and metastasis.

### miR-28-5p Directly Targets and Downregulates Rap1b Expression in Glioma Cells

#### miR-28-5p Expression Is Inversely Correlated With Rap1b mRNA and Protein Expression in Glioma Cells

**Figure 6:**
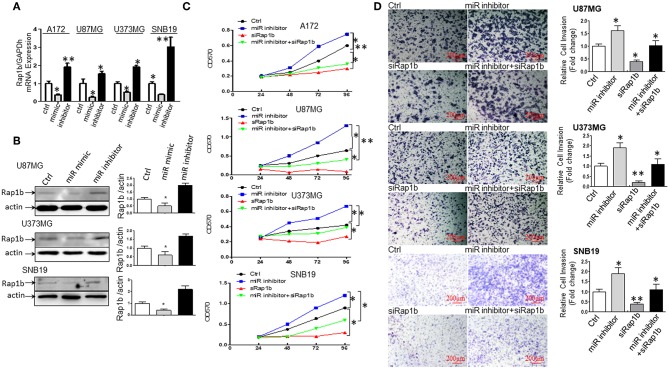
miR-28-5p directly targets and downregulates Rap1b expression in glioma cells. **(A,B)** miR-28-5p expression is inversely correlated with Rap1b mRNA and protein expression in glioma cells. We analyzed the expression of the endogenous Rap1b after transiently transfecting cells with either (a) miR-28-5p mimics; (b) miR-28-5p inhibitor; (c) control miR-28-5p-NC (ctrl) in A172, U87MG, U373MG, and SNB19 cells. The transient transfection efficiency was determined by qRT-PCR and Western blot. As expected, Rap1b mRNA expression levels were significantly reduced by miR-28-5p mimics and significantly enhanced by miR-28-5p inhibitor in each of the four glioma cell lines **(A)**. Similar, the Rap1b protein expressions displayed the same patterns as those of mRNA expressions in U87MG, U373MG, and SNB19 cells **(B)**. The densitometry analysis performed using the ImageQuant program are on the right side of each Western blot gel. **(C,D)** miR-28-5p inhibits glioma cell proliferation and invasion by directly targeting Rap1b. To further clarify whether or not tumor suppressive roles of miR-28-5p were dependent on Rap1b expression, 100 nM of siRap1b was co-transfected with 30 nM of miR28-5p inhibitor in A172, U87MG, U373MG, and SNB19 glioma cell lines to assess glioma cells' proliferation by MTT assay and evaluate glioma cell invasion by Matrigel transwell invasion assay. The reduction of Rap1b expression by siRap1b effectively reduced the miR-28-5p inhibitor-induced glioma cell proliferation **(C)**, and the miR-28-5p inhibitor-induced invasion (**D**, Photomicrographs were taken at 10x magnification) in each of the four glioma cell lines above. All data are from triplicate samples performed in two different independent experiments. (*P < 0.05, **P < 0.01).

To further explore the association between miR-28-5p and Rap1b in glioma cells, we analyzed the expression of the endogenous Rap1b after transiently transfecting cells with either (a) miR-28-5p mimics, (b) miR-28-5p inhibitor, or (c) control miR-28-5p-NC in A172, U87MG, U373MG, and SNB19 cells. The transient transfection efficiency was determined by qRT-PCR and Western blot. As shown in Figure 6A, Rap1b mRNA expression levels were significantly reduced by miR-28-5p mimics and significantly enhanced by miR-28-5p inhibitor in each of the four glioma cell lines. Similarly, the Rap1b protein expressions displayed the same patterns as those of mRNA expressions in U87MG, U373MG, and SNB19 cells (Figure 6B). These results confirm that miR-28-5p regulates both the expression levels of Rap1b mRNA and protein.

#### miR-28-5p Inhibits Glioma Cell Proliferation and Invasion by Directly Targeting Rap1b

To further clarify whether or not tumor suppressive roles of miR-28-5p were dependent on Rap1b expression, 100 nM of siRap1b was co-transfected with 30 nM of miR-28-5p inhibitor in A172, U87MG, U373MG, and SNB19 glioma cell lines to assess glioma cells' proliferation by MTT assay and glioma cell invasion by Matrigel transwell invasion assay. As expected, the reduction of Rap1b expression by siRap1b effectively reduced glioma cell proliferation induced by miR-28-5p inhibitor during 24 to 96 h post-transfection by a maximum of 52, 41.8, and 69.2% for A172, U87MG, and U373MG at 96 h, 60% for SNB19 at 48 h (Figure 6C). Similarly, siRap1b markedly reduced miR-28-5p inhibitor-induced invasion by 35.6, 42.1, and 39.0% in U87MG, U373MG, and SNB19 cells at 72 h, respectively (Figure 6D, ^*^P < 0.05, ^**^P < 0.01, one-way ANOVA). These results suggest that Rap1b is able to at least partially restore the function of miR-28-5p, and these functional restoration assays support that miR-28-5p functions upstream of Rap1b and is dependent on Rap1b.

## Discussion

In the current study, we utilized four glioma cell lines, A172, U87MG, U373MG, and SNB19, based on their different molecular characteristics. U373MG has a p53 gene mutation (33), while A172, U87MG (33), and SNB19 are PTEN-mutant cell lines (34, 35). In addition to harboring PTEN and CDKN2A (p16^INK4a^) mutation as in A172, U87MG has another CDKN2C (p18^INK4c^) mutation (36). Although U373MG cells may share common origins with SNB19, these two cell lines appear to have evolved to exhibit distinct karyotypes and drug sensitivities (37); thus, they serve as culture models with different molecular background for comparison in this study.

TRPM7 channel is nonselectively permeable to many divalent cations such as Ca^2+^, Mg^2+^, and Zn^2+^, which comprises the TRPM7-mediated inward current. In addition, it has the unique characteristic of α-kinase domain at its carboxyl-terminal (17). The kinase domain of TRPM7 is not essential for the activation of its channel, but structural changes in the kinase domain could alter the sensitivity of channel activation to divalent cation (17). TRPM7-dependent divalent cations, growth factors (GFs), hypoxia and/or other signals can open TRPM7 channel and increase cell proliferation. In this regard, the major activated signaling pathways activated include PI3K/AKT/mTOR (38–40), Src/MAPKs/JNK, p38, and ERK (41–45). Depleted TRPM7 resulted in the inactivation of STAT3 and the disruption of proliferation of thymocytes (46). On the other hand, the opening of TRPM7 channels causes a local increases in Ca^2+^/Mg^2+^ concentration, which affects the recruitment/targeting of TRPM7 kinase substrates (16). TRPM7-kinase phosphorylates downstream eEF2-k on Ser78 in human (Ser77 in mouse eEF2-k), causing increased stability of eEF2-k, strengthening inhibitory eEF2 at Thr56 phosphorylation and resulting in an increased translational efficiency when Mg^2+^ is available (16). The channel kinase of TRPM7 participates in the regulation of cell migration by modulating phosphorylation of annexin 1 (47–50), myosin IIA (51–53) and calpain (54) in response to various stimuli in tumor cells. In the present study, we evaluated TRPM7 channels' properties using a deletion mutant of human TRPM7 in which the entire kinase domain was removed following amino acid 1569 (TRPM7 Δkinase or M7-DK) and a mutant with a point mutation in the ATP binding site of the α-kinase domain (K1648R, or M7-KR). We found that the channel activity of TRPM7 is required for cell growth while the kinase domain is required for cell migration/invasion. This finding is important for designing and developing drugs targeting TRPM7 in human malignancies.

Among 2,588 mature and 1,881 precursor human miRNA sequences in 12 miRNA databases, 365 miRNAs were found to be deregulated in glioblastoma (55). The genes targeted by the deregulated miRNAs in glioblastoma include many pathways such as cell growth/proliferation, apoptosis, invasion and metastasis, angiogenesis, autophagy, and drug resistance (56). miR-28-5p was found to interfere with genes involved in cell replication and cell cycle checkpoints (57). In glioblastoma, the role of miR-28-5p has not yet been fully explored, but its structurally related miR-708 was demonstrated to inhibit glioblastoma cell proliferation by targeting EZH2, AKT1, MMP2, CCND1, Parp-1, and Bcl-2 (58). Our results suggest that miRNA changes demonstrate different glioma tumorigenicity, and miR-26b-5p, miR-4530, and miR-28-5p are regulated by TRPM7 and participate in glioma progression. In the present study, we further elucidated the TRPM7/miR-28-5p/Rap1b axis in gliomagenesis using multiple cell lines with different mutational status. Our results showed that miR-28-5p inhibits glioma cell proliferation and invasion by targeting its downstream Rap1b gene, and miR-28-5p is regulated by TRPM7.

Functional analyses of miR-28-5p revealed tumor suppressive properties caused by the inhibition of Rap1b, E2F6, IGF-1, IL-34, and AKT. A study reported that miR-28-5p inhibited cell proliferation and migration by directly suppressing Rap1b gene in renal cell carcinoma (31). In prostate cancer, the miR-28-5p targeted E2F6 and induced apoptosis in DU-145 cells (7). The ectopic miR-28-5p expression downregulates insulin-like growth factor 1 (IGF1) protein and the expression of miR-28-5p correlates negatively with IGF1 protein level in hepatocellular carcinoma (HCC) cells, which indicate that miR-28-5p-IGF1-PI3K/AKT pathway may play an important role in the development of HCC (10). IL-34 is a direct downstream target of miR-28-5p whose expression is inversely correlated with IL-34 expression (30). Lastly, miR-28-5p may act as a tumor suppressor gene, which inhibited the invasion and metastasis of gastric cancer by inhibiting the activation of the AKT signaling pathway (8). By contrast, miR-28 also served as a tumor promoting factor in some specific tumors. For instance, it is a thrombopoietin receptor (TpoR, MPL) targeting miRNA, which is overexpressed in the platelets of patients with myeloproliferative neoplasms (MPNs) and its negative role in megakaryocyte differentiation leads to MPNs through the downregulation of MPL (59). In the process of ovarian cancer development and progression, miR-28-5p downregulates N4BP1, forces cancer cells to enter S phase, and promotes ovarian cancer cell proliferation and invasion (29). Taken together, miR-28-5p can function as either tumor suppressor genes or oncogenes, reflecting cell type-specific and tissue-specific roles of this miRNA.

miRNAs are involved in diverse biological processes by binding to different regions of the target mRNA sequences, such as the 3′-untranslated regions, coding sequences, or 5′-untranslated regions. The same hairpin RNA structure can produce mature miRNAs from each strand, named 5p and 3p, that can bind to different mRNAs (60). Almeida et al. (60) found that strand-specific 5p and 3p of miR-28-5 have distinct functions in colorectal cancer cells, where miR-28-5p suppressed cell proliferation, resulting in apoptosis and G1 arrest in the cell cycle, while miR-28-3p had no influence on proliferation *in vitro*. In addition, miR-28-5p and miR-28-3p had opposite effects on migration and invasion *in vitro*, with miR-28-5p decreasing cell migration and miR-28-3p increasing cell migration, which appears to be independent of cell growth. Overall, miR-28-3p overrides miR-28-5p to promote colorectal cancer cell metastases *in vivo*. The contrasting biological effects caused by miR-28-5p and miR-28-3p might be partly due to their binding to different targets where miR-28-5p targets CCND1 and HoxB3 while miR-28-3p targets Nm23-H1. In HCC, Kaplan-Meier's analysis showed that HCC patients with miR-28-5p overexpression, but not with miR-28-3p overexpression, had better overall survival rate. Girardot et al. (59) identified two miRNAs that are closely related to miR-28. miR-151 and miR-28 share 80% sequence identity, while miR-708 share 68% sequence identity with miR-28 and 71% with miR-151. All three miRNAs targeted the MPL 3′ UTR for translational inhibition. However, the combination of miR-28 with miR-151 and miR-708 did not produce synergic inhibition due to all targeting the same sequence in the 3′UTR of MPL.

The normal physiological role of the small GTP-binding protein Rap1 is to antagonize Ras mitogenic signals. However, in 1998, Altschuler et al. first revealed that Rap1 is a conditional oncoprotein (61). Later, Rap1 was demonstrated to be required for pancreatic and prostate cancer cell metastasis and angiogenesis but not for the proliferation properties of these cancer cells (62). In 2014, Sayyah's group published the first study showing that this small G-protein is required for glioblastoma cell growth *in vitro* by activating downstream signaling of G-protein-coupled receptor and RhoA utilizing Rap1 knockdown techniques. However, they stated that this critical role was caused by the isoform Rap1a, not Rap1b (32). Rap1a and Rap1b are 95% homologous, but differences in subcellular localization of two isoforms and their mechanisms of activation resulted in their distinctive functions and roles in glioblastoma cell proliferation (63, 64). In this study, we found Rap1b demonstrated the mitogenic roles in both glioma cell proliferation and invasion.

A positive correlation has been shown between TRPM7 and Rap1b in Figure 4A. There are several possible mechanistic links between the two proteins. First, they may connect to each other by miR-28-5p. In this study, we showed that TRPM7 downregulated miR-28-5p, while miR-28-5p downregulated Rap1b; this is likely one mechanism of a positive correlation between TRPM7 and miR-28-5p. Second, TRPM7 and Rap1b may be connected by Notch signaling. In hematopoietic stem cell development, Integrin-mediated cell adhesion was promoted by Notch-regulated Rap1b signaling (65); while our published data also demonstrated that TRPM7 regulated the Notch pathway in gliomagenesis (1).

Taken together, the discovery of cellular and molecular targets modulated by the tumor suppressor miR-28-5p and its upstream regulator TRPM7 molecule provides key insights into the potential mechanisms of glioma tumorigenesis and suggests novel therapeutic targets for glioma treatments.

## Data Availability Statement

The datasets generated for this study are available on request to the corresponding author.

## Author Contributions

SG, GW, and ML designed the study protocol. JW and AG performed experiments based on glioma cell cultures and evaluated the data with the help of ML. ML performed the biostatistical evaluation of the data. AG and ML wrote the manuscript with contributions and final approval by all authors. IC, SG, JH, and GW contributed to the critical reading and revision of the manuscript.

### Conflict of Interest

The authors declare that the research was conducted in the absence of any commercial or financial relationships that could be construed as a potential conflict of interest.

## References

[1] Liu M Inoue K Leng T Guo S Xiong ZG. TRPM7 channels regulate glioma stem cell through STAT3 and Notch signaling pathways. Cell Signal. (2014) 26:2773–81. 10.1016/j.cellsig.2014.08.020 25192910 PMC4405379

[2] Miyamoto K Seki N Matsushita R Yonemori M Yoshino H Nakagawa M. Tumour-suppressive miRNA-26a-5p and miR-26b-5p inhibit cell aggressiveness by regulating PLOD2 in bladder cancer. Br J Cancer. (2016) 115:354–63. 10.1038/bjc.2016.179 27310702 PMC4973152

[3] Bartel DP. MicroRNAs: genomics, biogenesis, mechanism, and function. Cell. (2004) 116:281–97. 10.1016/S0092-8674(04)00045-5 14744438

[4] Carthew RW Sontheimer EJ. Origins and mechanisms of miRNAs and siRNAs. Cell. (2009) 136:642–55. 10.1016/j.cell.2009.01.035 19239886 PMC2675692

[5] Di Leva G Croce CM. Roles of small RNAs in tumor formation. Trends Mol Med. (2010) 16:257–67. 10.1016/j.molmed.2010.04.001 20493775 PMC2885513

[6] Malzkorn B Wolter M Liesenberg F Grzendowski M Stuhler K Meyer HE. Identification and functional characterization of microRNAs involved in the malignant progression of gliomas. Brain Pathol. (2010) 20:539–50. 10.1111/j.1750-3639.2009.00328.x 19775293 PMC8094849

[7] Rizzo M Berti G Russo F Evangelista M Pellegrini M Rainaldi G. The miRNA pull out assay as a method to validate the miR-28–5p targets identified in other tumor contexts in prostate cancer. Int J Genomics. (2017) 2017:5214806. 10.1155/2017/5214806 29085832 PMC5632462

[8] Xiao F Cheng Z Wang P Gong B Huang H Xing Y. MicroRNA-28–5p inhibits the migration and invasion of gastric cancer cells by suppressing AKT phosphorylation. Oncol Lett. (2018) 15:9777–85. 10.3892/ol.2018.8603 29928352 PMC6004724

[9] Liu J Liu XQ Liu Y Sun YN Li S Li CM. MicroRNA 28–5p regulates ATP-binding cassette transporter A1 via inhibiting extracellular signal-regulated kinase 2. Mol Med Rep. (2016) 13:433–40. 10.3892/mmr.2015.4563 26718613

[10] Shi X Teng F. Down-regulated miR-28–5p in human hepatocellular carcinoma correlated with tumor proliferation and migration by targeting insulin-like growth factor-1 (IGF-1). Mol Cell Biochem. (2015) 408:283–93. 10.1007/s11010-015-2506-z 26160280

[11] Thuringer D Chanteloup G Winckler P Garrido C. The vesicular transfer of CLIC1 from glioblastoma to microvascular endothelial cells requires TRPM7. Oncotarget. (2018) 9:33302–11. 10.18632/oncotarget.26048 30279961 PMC6161795

[12] Sander P Mostafa H Soboh A Schneider JM Pala A Baron AK. Vacquinol-1 inducible cell death in glioblastoma multiforme is counter regulated by TRPM7 activity induced by exogenous ATP. Oncotarget. (2017) 8:35124–37. 10.18632/oncotarget.16703 28410232 PMC5471040

[13] Chen HS Lu AQ Yang PY Liang J Wei Y Shang YW. MicroRNA-28–5p regulates glioma cell proliferation, invasion and migration by targeting SphK1. Eur Rev Med Pharmacol Sci. (2019) 23:6621–8. 10.26355/eurrev_201908_18551 31378904

[14] She X Yu Z Cui Y Lei Q Wang Z Xu G. miR-128 and miR-149 enhance the chemosensitivity of temozolomide by Rap1B-mediated cytoskeletal remodeling in glioblastoma. Oncol Rep. (2014) 32:957–64. 10.3892/or.2014.3318 25017996

[15] Malchinkhuu E Sato K Maehama T Ishiuchi S Yoshimoto Y Mogi C. Role of Rap1B and tumor suppressor PTEN in the negative regulation of lysophosphatidic acid–induced migration by isoproterenol in glioma cells. Mol Biol Cell. (2009) 20:5156–65. 10.1091/mbc.e09-08-0692 19864456 PMC2793292

[16] Perraud AL Zhao X Ryazanov AG Schmitz C. The channel-kinase TRPM7 regulates phosphorylation of the translational factor eEF2 via eEF2-k. Cell Signal. (2011) 23:586–93. 10.1016/j.cellsig.2010.11.011 21112387 PMC3038675

[17] Schmitz C Perraud AL Johnson CO Inabe K Smith MK Penner R. Regulation of vertebrate cellular Mg2+ homeostasis by TRPM7. Cell. (2003) 114:191–200. 10.1016/S0092-8674(03)00556-7 12887921

[18] Larco DO Semsarzadeh NN Cho-Clark M Mani SK Wu TJ. β-Arrestin 2 Is a mediator of GnRH-(1–5) signaling in immortalized GnRH neurons. Endocrinology. (2013) 154:4726–36. 10.1210/en.2013-1286 24140715

[19] Liu M Wilson NO Hibbert JM Stiles JK. STAT3 regulates MMP3 in heme-induced endothelial cell apoptosis. PLoS ONE. (2013) 8:e71366. 10.1371/journal.pone.0071366 23967200 PMC3742773

[20] Haddock AN Labuzan SA Haynes AE Hayes CS Kakareka KM Waddell DS. Dual-specificity phosphatase 4 is upregulated during skeletal muscle atrophy and modulates extracellular signal-regulated kinase activity. AJP Cell Physiology. (2019) 316:C567–81. 10.1152/ajpcell.00234.2018 30758994

[21] Varkonyi-Gasic E Wu R Wood M Walton EF Hellens RP. Protocol: a highly sensitive RT-PCR method for detection and quantification of microRNAs. Plant Methods. (2007) 3:12. 10.1186/1746-4811-3-12 17931426 PMC2225395

[22] Chen C Ridzon DA Broomer AJ Zhou Z Lee DH Nguyen JT. Real-time quantification of microRNAs by stem-loop RT-PCR. Nucleic Acids Res. (2005) 33:e179. 10.1093/nar/gni178 16314309 PMC1292995

[23] Balcells I Cirera S Busk PK. Specific and sensitive quantitative RT-PCR of miRNAs with DNA primers. BMC Biotechnol. (2011) 11:70. 10.1186/1472-6750-11-70 21702990 PMC3135530

[24] Busk PK. A tool for design of primers for microRNA-specific quantitative RT-qPCR. BMC Bioinformatics. (2014) 15:29. 10.1186/1471-2105-15-29 24472427 PMC3922658

[25] Czimmerer Z Hulvely J Simandi Z Varallyay E Havelda Z Szabo E. A versatile method to design stem-loop primer-based quantitative PCR assays for detecting small regulatory RNA molecules. PLoS ONE. (2013) 8:e55168. 10.1371/journal.pone.0055168 23383094 PMC3561390

[26] Kang ST Hsieh YS Feng CT Chen YT Yang PE Chen WM. miPrimer: an empirical-based qPCR primer design method for small noncoding microRNA. RNA. (2018) 24:304–12. 10.1261/rna.061150.117 29208706 PMC5824350

[27] Marcial-Quino J Gomez-Manzo S Fierro F Vanoye-Carlo A Rufino-Gonzalez Y Sierra-Palacios E. Stem-loop RT-qPCR as an efficient tool for the detection and quantification of small RNAs in giardia lamblia. Genes. (2016) 7:131. 10.3390/genes7120131 27999395 PMC5192507

[28] Liu Y Beyer A Aebersold R. On the dependency of cellular protein levels on mRNA abundance. Cell. (2016) 165:535–50. 10.1016/j.cell.2016.03.014 27104977

[29] Xu J Jiang N Shi H Zhao S Yao S Shen H. miR-28–5p promotes the development and progression of ovarian cancer through inhibition of N4BP1. Int J Oncol. (2017) 50:2236. 10.3892/ijo.2017.3977 28498450 PMC5435330

[30] Zhou SL Hu ZQ Zhou ZJ Dai Z Wang Z Cao Y. miR-28–5p-IL-34-macrophage feedback loop modulates hepatocellular carcinoma metastasis. Hepatology. (2016) 63:1560–75. 10.1002/hep.28445 26754294

[31] Wang C Wu C Yang Q Ding M Zhong J Zhang CY. miR-28–5p acts as a tumor suppressor in renal cell carcinoma for multiple antitumor effects by targeting RAP1B. Oncotarget. (2016) 7:73888–902. 10.18632/oncotarget.12516 27729617 PMC5342021

[32] Sayyah J Bartakova A Nogal N Quilliam LA Stupack DG Brown JH. The Ras-related protein, Rap1A, mediates thrombin-stimulated, integrin-dependent glioblastoma cell proliferation and tumor growth. J Biol Chem. (2014) 289:17689–98. 10.1074/jbc.M113.536227 24790104 PMC4067203

[33] Ishii N Maier D Merlo A Tada M Sawamura Y Diserens AC. Frequent co-alterations of TP53, p16/CDKN2A, p14ARF, PTEN tumor suppressor genes in human glioma cell lines. Brain Pathol. (1999) 9:469–79. 10.1111/j.1750-3639.1999.tb00536.x 10416987 PMC8098486

[34] Chakrabarti M Ray SK. Synergistic anti-tumor actions of luteolin and silibinin prevented cell migration and invasion and induced apoptosis in glioblastoma SNB19 cells and glioblastoma stem cells. Brain Res. (2015) 1629:85–93. 10.1016/j.brainres.2015.10.010 26471408

[35] Memmel S Sukhorukov VL Horing M Westerling K Fiedler V Katzer A. Cell surface area and membrane folding in glioblastoma cell lines differing in PTEN and p53 status. PLoS ONE. (2014) 9:e87052. 10.1371/journal.pone.0087052 24498019 PMC3909012

[36] Allen M Bjerke M Edlund H Nelander S Westermark B. Origin of the U87MG glioma cell line: good news and bad news. Sci Transl Med. (2016) 8:354re3. 10.1126/scitranslmed.aaf6853 27582061

[37] Stepanenko AA Kavsan VM. Karyotypically distinct U251, U373, and SNB19 glioma cell lines are of the same origin but have different drug treatment sensitivities. Gene. (2014) 540:263–5. 10.1016/j.gene.2014.02.053 24583163

[38] Sahni J Scharenberg AM. TRPM7 ion channels are required for sustained phosphoinositide 3-kinase signaling in lymphocytes. Cell Metab. (2008) 8:84–93. 10.1016/j.cmet.2008.06.002 18590694 PMC3199037

[39] Sahni J Tamura R Sweet IR Scharenberg AM. TRPM7 regulates quiescent/proliferative metabolic transitions in lymphocytes. Cell Cycle. (2010) 9:3565–74. 10.4161/cc.9.17.12798 20724843 PMC3047620

[40] Zhan F Barlogie B Arzoumanian V Huang Y Williams DR Hollmig K. Gene-expression signature of benign monoclonal gammopathy evident in multiple myeloma is linked to good prognosis. Blood. (2007) 109:1692–700. 10.1182/blood-2006-07-037077 17023574 PMC1794073

[41] Davis FM Azimi I Faville RA Peters AA Jalink K Putney JW Jr. Induction of epithelial-mesenchymal transition (EMT) in breast cancer cells is calcium signal dependent. Oncogene. (2013) 33:2307–16. 10.1038/onc.2013.187 23686305 PMC3917976

[42] Desai BN Krapivinsky G Navarro B Krapivinsky L Carter BC Febvay S. Cleavage of TRPM7 releases the kinase domain from the ion channel and regulates its participation in Fas-induced apoptosis. Dev Cell. (2012) 22:1149–62. 10.1016/j.devcel.2012.04.006 22698280 PMC3397829

[43] Inoue K Xiong ZG. Silencing TRPM7 promotes growth/proliferation and nitric oxide production of vascular endothelial cells via the ERK pathway. Cardiovasc Res. (2009) 83:547–57. 10.1093/cvr/cvp153 19454490 PMC2709465

[44] Meng X Cai C Wu J Cai S Ye C Chen H. TRPM7 mediates breast cancer cell migration and invasion through the MAPK pathway. Cancer Lett. (2013) 333:96–102. 10.1016/j.canlet.2013.01.031 23353055

[45] Su LT Chen HC Gonzalez-Pagan O Overton JD Xie J Yue L. TRPM7 activates m-calpain by stress-dependent stimulation of p38 MAPK and c-Jun N-terminal kinase. J Mol Biol. (2010) 396:858–69. 10.1016/j.jmb.2010.01.014 20070945 PMC2825087

[46] Jin J Desai BN Navarro B Donovan A Andrews NC Clapham DE. Deletion of Trpm7 disrupts embryonic development and thymopoiesis without altering Mg2+ homeostasis. Science. (2008) 322:756–60. 10.1126/science.1163493 18974357 PMC2605283

[47] Dorovkov MV Kostyukova AS Ryazanov AG. Phosphorylation of annexin A1 by TRPM7 kinase: a switch regulating the induction of an alpha-helix. Biochemistry. (2011) 50:2187–93. 10.1021/bi101963h 21280599 PMC3062375

[48] Gerke V Moss SE. Annexins: from structure to function. Physiol Rev. (2002) 82:331–71. 10.1152/physrev.00030.2001 11917092

[49] Rescher U Gerke V. Annexins–unique membrane binding proteins with diverse functions. J Cell Sci. (2004) 117:2631–9. 10.1242/jcs.01245 15169834

[50] Rothhut B. Participation of annexins in protein phosphorylation. Cell Mol Life Sci. (1997) 53:522–6. 10.1007/s000180050066 9230930 PMC11147422

[51] Clark K Langeslag M van Leeuwen B Ran L Ryazanov AG Figdor CG. TRPM7, a novel regulator of actomyosin contractility and cell adhesion. EMBO J. (2006) 25:290–301. 10.1038/sj.emboj.7600931 16407977 PMC1383514

[52] Langeslag M Clark K Moolenaar WH van Leeuwen FN Jalink K. Activation of TRPM7 channels by phospholipase C-coupled receptor agonists. J Biol Chem. (2007) 282:232–9. 10.1074/jbc.M605300200 17095511

[53] Nakasawa T Takahashi M Matsuzawa F Aikawa S Togashi Y Saitoh T. Critical regions for assembly of vertebrate nonmuscle myosin II. Biochemistry. (2005) 44:174–83. 10.1021/bi048807h 15628858

[54] Su LT Agapito MA Li M Simonson WT Huttenlocher A Habas R. TRPM7 regulates cell adhesion by controlling the calcium-dependent protease calpain. J Biol Chem. (2006) 281:11260–70. 10.1074/jbc.M512885200 16436382 PMC3225339

[55] Moller HG Rasmussen AP Andersen HH Johnsen KB Henriksen M Duroux M. A systematic review of microRNA in glioblastoma multiforme: micro-modulators in the mesenchymal mode of migration and invasion. Mol Neurobiol. (2013) 47:131–44. 10.1007/s12035-012-8349-7 23054677 PMC3538124

[56] Banelli B Forlani A Allemanni G Morabito A Pistillo MP Romani M. MicroRNA in glioblastoma: an overview. Int J Genomics. (2017) 2017:7639084. 10.1155/2017/7639084 29234674 PMC5695025

[57] Hell MP Thoma CR Fankhauser N Christinat Y Weber TC Krek W. miR-28–5p promotes chromosomal instability in VHL-associated cancers by inhibiting Mad2 translation. Cancer Res. (2014) 74:2432–43. 10.1158/0008-5472.CAN-13-2041 24491803

[58] Guo P Lan J Ge J Nie Q Mao Q Qiu Y. miR-708 acts as a tumor suppressor in human glioblastoma cells. Oncol Rep. (2013) 30:870–6. 10.3892/or.2013.2526 23754151

[59] Girardot M Pecquet C Boukour S Knoops L Ferrant A Vainchenker W. miR-28 is a thrombopoietin receptor targeting microRNA detected in a fraction of myeloproliferative neoplasm patient platelets. Blood. (2010) 116:437–45. 10.1182/blood-2008-06-165985 20445018

[60] Almeida MI Nicoloso MS Zeng L Ivan C Spizzo R Gafa R. Strand-specific miR-28–5p and miR-28–3p have distinct effects in colorectal cancer cells. Gastroenterology. (2012) 142:886–96.e9. 10.1053/j.gastro.2011.12.047 22240480 PMC3321100

[61] Altschuler DL Ribeiro-Neto F. Mitogenic and oncogenic properties of the small G protein Rap1b. Proc Natl Acad Sci USA. (1998) 95:7475–9. 10.1073/pnas.95.13.7475 9636174 PMC22655

[62] Huang M Anand S Murphy EA Desgrosellier JS Stupack DG Shattil SJ. EGFR-dependent pancreatic carcinoma cell metastasis through Rap1 activation. Oncogene. (2012) 31:2783–93. 10.1038/onc.2011.450 21963850 PMC3711644

[63] Severson EA Lee WY Capaldo CT Nusrat A Parkos CA. Junctional adhesion molecule A interacts with Afadin and PDZ-GEF2 to activate Rap1A, regulate beta1 integrin levels, and enhance cell migration. Mol Biol Cell. (2009) 20:1916–25. 10.1091/mbc.e08-10-1014 19176753 PMC2663925

[64] Wittchen ES Aghajanian A Burridge K. Isoform-specific differences between Rap1A and Rap1B GTPases in the formation of endothelial cell junctions. Small GTPases. (2011) 2:65–76. 10.4161/sgtp.2.2.15735 21776404 PMC3136906

[65] Rho SS Kobayashi I Oguri-Nakamura E Ando K Fujiwara M Kamimura N. Rap1b promotes notch-signal-mediated hematopoietic stem cell development by enhancing integrin-mediated cell adhesion. Dev Cell. (2019) 49:681–96.e6. 10.1016/j.devcel.2019.03.023 31006651

